# Using machine learning to understand neuromorphological change and image‐based biomarker identification in Cavalier King Charles Spaniels with Chiari‐like malformation‐associated pain and syringomyelia

**DOI:** 10.1111/jvim.15621

**Published:** 2019-09-24

**Authors:** Michaela Spiteri, Susan P. Knowler, Clare Rusbridge, Kevin Wells

**Affiliations:** ^1^ CVSSP, Centre for Vision, Speech and Signal Processing University of Surrey Guildford United Kingdom; ^2^ Faculty of Health & Medical Sciences School of Veterinary Medicine Guildford United Kingdom; ^3^ Fitzpatrick Referrals Orthopaedics and Neurology Godalming United Kingdom

**Keywords:** biomarker, brachycephaly, canine, Cavalier King Charles Spaniel, craniosynostosis, image registration, machine learning, MRI, radiology and diagnostic imaging

## Abstract

**Background:**

Chiari‐like malformation (CM) is a complex malformation of the skull and cranial cervical vertebrae that potentially results in pain and secondary syringomyelia (SM). Chiari‐like malformation‐associated pain (CM‐P) can be challenging to diagnose. We propose a machine learning approach to characterize morphological changes in dogs that may or may not be apparent to human observers. This data‐driven approach can remove potential bias (or blindness) that may be produced by a hypothesis‐driven expert observer approach.

**Hypothesis/Objectives:**

To understand neuromorphological change and to identify image‐based biomarkers in dogs with CM‐P and symptomatic SM (SM‐S) using a novel machine learning approach, with the aim of increasing the understanding of these disorders.

**Animals:**

Thirty‐two client‐owned Cavalier King Charles Spaniels (CKCSs; 11 controls, 10 CM‐P, 11 SM‐S).

**Methods:**

Retrospective study using T2‐weighted midsagittal Digital Imaging and Communications in Medicine (DICOM) anonymized images, which then were mapped to images of an average clinically normal CKCS reference using Demons image registration. Key deformation features were automatically selected from the resulting deformation maps. A kernelized support vector machine was used for classifying characteristic localized changes in morphology.

**Results:**

Candidate biomarkers were identified with receiver operating characteristic curves with area under the curve (AUC) of 0.78 (sensitivity 82%; specificity 69%) for the CM‐P biomarkers collectively and an AUC of 0.82 (sensitivity, 93%; specificity, 67%) for the SM‐S biomarkers, collectively.

**Conclusions and clinical importance:**

Machine learning techniques can assist CM/SM diagnosis and facilitate understanding of abnormal morphology location with the potential to be applied to a variety of breeds and conformational diseases.

**Abbreviations**
AUC: area under the curve
CKCS: Cavalier King Charles Spaniel
CM: Chiari‐like malformation
CM‐N: control dogs: no SM no CM pain; 4 years of age and older
CM‐P: pain associated with Chiari‐like malformation
CSF: cerebrospinal fluid
DICOM: Digital Imaging and Communications in Medicine
FPR: false‐positive rate (also known as 1 − specificity)
ICC: intraclass correlation coefficient
MRI: magnetic resonance imaging
PCA: principal component analysis
ROC: receiver operating characteristic
SFFS: sequential floating forward selection
SM: syringomyelia
SM‐S: syringomyelia and associated clinical signs
SVM: support vector machine
TPR: true‐positive rate (also known as sensitivity)

## 1 INTRODUCTION

Syringomyelia (SM) is characterized by the development of fluid‐filled cavities within the spinal cord. The pathogenesis of SM is debated, but there is consensus that it is associated with obstruction of cerebrospinal fluid (CSF) channels, especially when that obstruction is at the craniocervical junction and foramen magnum. In dogs, SM most commonly is associated with Chiari‐like malformation (CM),^1^, ^2^, ^3^, ^4^ a complex developmental malformation of the skull and cranial cervical vertebrae characterized by rostrocaudal bony insufficiency resulting in conformational changes and overcrowding of the brain and cervical spinal cord, particularly at the craniocervical junction.

Depending on the site and extent of spinal cord damage, SM may result in behavioral signs of pain, phantom (fictive) scratching, scoliosis, weakness, and sensory deficits although some dogs may be asymptomatic, especially if the syrinx is narrow and centrally located.^5^ Brachycephalic toy breeds are predisposed to CM and SM, especially the Cavalier King Charles Spaniel (CKCS)^1^, ^4^, ^6^ in which there is a hereditary predisposition.^1^, ^4^

Diagnosis of CM and SM requires magnetic resonance imaging (MRI). Whereas identification of SM is straightforward, assessment of CM is more difficult. Originally described in 2000 as a small‐volume caudal fossa with cerebellar herniation,^7^ numerous studies have now shown that the condition is more complex.^1^, ^4^, ^8^, ^9^, ^10^, ^11^, ^12^, ^13^, ^14^, ^15^ In addition to hypoplasia of the supra‐ and basioccipital bones, resulting in short cranial base, decreased caudal fossa volume, and tendency for cerebellar herniation, the condition is associated with a compensatory increase in the height of the cranial fossa with decreased occipital crest,^1^ rostral displacement of the atlas and axis (atlanto‐occipital overlapping), medulla oblongata elevation with or without kinking, more acute angulation of the axis bone to the cranial base (cervical flexure), more acute angle at the spheno‐occipital synchondrosis (sphenoid flexure),^1^, ^2^ a relatively large cerebellum,^16^, ^17^, ^18^ decreased volume of jugular foramen and venous sinus, and dorsal compression of the spinal cord by atlantoaxial bands.^19^, ^20^ Indeed, most CKCS have the condition^14^, ^21^ to some extent, but ascertaining severity and therefore risk of clinical disease and of passing on that risk to offspring has proved more problematic. Morphometric mapping can be used as a diagnostic tool for qualifying pain associated with CM (CM‐P). However, the process is laborious and requires specialist training and practice and currently is confined to research groups investigating the pathogenesis and for genetic studies.^4^

Traditional morphometric studies also have the potential disadvantage that they are hypothesis‐driven (ie, the researcher proposes the measurement to be tested based on prior knowledge or intuition). Our proposed method is based on a purely data‐driven approach. In other words, we propose to let the data “speak” in an unbiased way. This is facilitated by using machine learning methods to discover underlying patterns in the image data, which may or may not be immediately obvious, and that are not guided by human intervention (other than by labelling the fundamental phenotype). Thus, our aim was to identify potential imaging biomarkers of SM‐ and CM‐associated pain within the entire cranium.

## 2 MATERIALS AND METHODS

**Figure 1:**
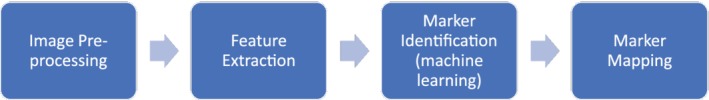
Schematic diagram showing the key steps involved in the proposed image analysis pipeline

The methodology was divided into 4 main sections, namely image preprocessing, feature extraction, biomarker identification using machine learning, and biomarker mapping (Figure 1). This pipeline was established with the aim of identifying specific locations within the brain related to CM‐P and clinically relevant SM in dogs that are different in some way to the features seen in control dogs.

**Figure 2:**
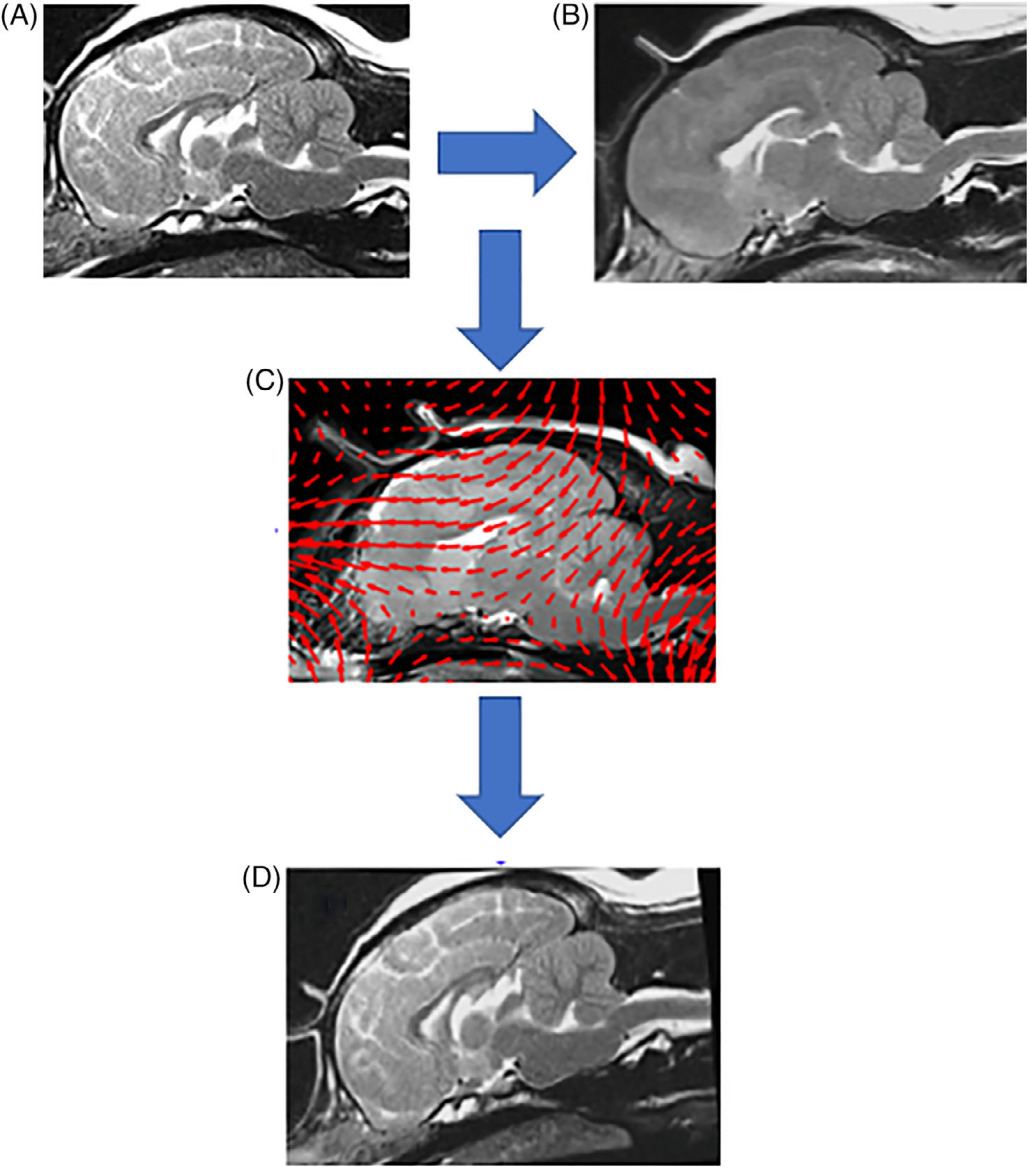
A schematic diagram showing the image registration process between 2 T2‐weighted cranial MRI mid‐sagittal slices: A, an MR image of an arbitrary dog in the dataset (query dog) prior to registration; B, an MR image of the reference image; C, the resultant movement or warp field for the pixels in the arbitrary image superimposed in red and plotted at every 10 pixels for clarity; D, the resultant query image following registration to the reference dog. This is based on the hypothesis that the deformation field or “pattern” that warps the query image onto the reference image illustrated in C can be used to discriminate between dogs with syringomyelia (SM), assumed to have greater levels of distortion (ie, pulling and twisting of the image) due to greater internal differences in morphology, compared to those in the control group

The method starts by using an image registration step. Image registration is a process sometimes referred to as mapping, morphing, or warping to align the subject in an image of interest onto another image. Furthermore, performing this step accommodates differences in head size that may occur as a consequence of sex, neuter status, and age. In the methodology described here, we selected an average control subject as our reference image and then registered dogs either in our diseased group or in the control group onto this reference image. The registration process produces a warp field or deformation image, which illustrates the displacement of each pixel in our clinically affected or control images when these are mapped or registered onto our reference subject (Figure 2). We then use machine learning methods to extract the key features, as discovered by the machine, rather than a human, which characterizes the differences in the way the images of dogs in our disease groups are deformed compared to our control group. Learning how to best distinguish, or separate, the 2 classes of dogs (diseased, control) given the selected image features is referred to as classification. The quality of this separation or classification then is evaluated using receiver operating characteristic (ROC) methods to estimate the sensitivity and specificity of the proposed approach.

We then can map these features back to the original images to allow us to infer the key locations in the brain that are deformed in diseased dogs compared with our control dogs, in order to identify the locations of potential image‐based biomarkers for the target disease.

The investigation was carried out on a data set compiled from midsagittal T2‐weighted head and cranial cervical MRI obtained from 32 CKCS dogs, imaged using the same MRI machine. Demographical data of the total cohort are (32; 21 male, 11 female) summarized as follows:

1. CM‐N (control): no SM, no CM‐P; ≥4 years of age (n = 10);
2. CM‐P: no SM with clinical and behavioral signs or both of pain associated with CM‐P^22^, ^23^; ≥4 years of age (n = 11);
3. SM‐S: clinically severe SM with syrinx transverse diameter ≥4 mm^22^ and clinical signs relating to the syrinx (eg, phantom scratching, scoliosis, paresis, proprioceptive deficits); all ages (n = 11).

The diagnosis of CM‐P is suggested in predisposed breeds presenting with multiple signs suggesting pain such as a history of vocalization without obvious trigger, when shifting position, when recumbent, and when being lifted under the sternum to a height; spinal pain; head and ear rubbing or scratching; refusal or difficulty to jump or use stairs; exercise intolerance or decreased activity; sleep disruption; or behavioral change described as becoming more anxious, aggressive, or withdrawn.^22^ These were considered in addition to the following MRI changes: effacement of the cranial subarachnoid space evidenced by decreased definition of the sulci filled with high signal CSF, variable ventriculomegaly, brachycephaly with shortening of the basicranium and prephenoid bone with decreased and more ventrally orientated olfactory bulbs, rostral forebrain flattening, rostrotentorial neuroparenchyma displaced dorsocaudally giving increased height to the cranium and decreasing the functional caudotentorial space and contributing to hindbrain herniation, and atlas closer to the skull with flattened supraoccipital bone. The diagnosis, however, is dependent on other causes of skin disease and ear disease and spinal pain being excluded and the dogs responding to analgesia that did not include corticosteroids and not having developed any other cause of pain or neurological dysfunction over a minimum of an 18‐month follow‐up period.

The value of ≥4 mm as a clinically relevant syrinx was decided on the basis of a previous study that identified this measurement as the cutoff for SM‐specific clinical signs.^22^

### 2.1 Image preprocessing

The process is initiated by identifying a control reference dog (ie, a typical CKCS unaffected by CM‐P and SM) by using all of the imaging features of the control group extracted in previous studies.^1^, ^24^ The mean value for each feature was computed for all unaffected CKCS in the control group. For each of these subjects, the deviation from the mean value for each feature was calculated and the sum of these deviations computed. The dog resulting in the smallest sum of deviations was chosen as the dog with the most average‐looking appearance in the control group and was referred to as the reference dog. The MR image of this average dog was used as a reference image throughout the study pipeline. The next step was to carry out image registration to align all of the images in the data set to the common reference dog, so as to facilitate comparison of the 2 separate classes on a pixel‐by‐pixel basis. Before registration, the MR images were cropped to ensure that only the soft tissues associated with the head were considered for analysis. The midline sagittal image was considered in each case, such that the study was carried out in 2‐dimensional space. Although the basic topological shape of each subject is similar, the use of different scanners may give rise to differences in pixel dimensions. Furthermore, the dogs were likely to have been setup at slightly different orientations during image acquisition.

### 2.2 Image registration

In order to spatially align each image into a single coordinate space, an initial affine image registration step was used (affine registration refers to a process that only applies translation, rotation, and shear to an image to align it with another image). This provides an approximate alignment, eliminates volumetric differences caused by use of different scanners, and minimizes any effects caused by setup errors during the MRI acquisition process.

After affine registration, the morphological differences between each dog and the clinically unaffected reference dog were obtained using an adaptation of the Demons nonrigid registration method.^25^, ^26^ Such a nonrigid registration allows the machine to “squeeze and squash” or “pull and twist” or both a target object to best align it with the reference subject in the reference image. This produces a deformed query image, which is spatially aligned to the reference image, as well as a deformation matrix, or deformation field, that describes the distortions or deformations produced by image registration when mapping and aligning 1 image to another. The deformation matrix maps each pixel from a query image to a corresponding pixel on the reference dog image. This process is repeated for all of the subjects in the data set, enabling the cohort to be compared on a pixel‐by‐pixel basis. This process is illustrated in Figure 2. Afterward, an attempt is made to extract and select a set of features that characterizes the distortions seen in the warp field images for the 2 groups of dogs (control and diseased).

### 2.3 Feature selection

Feature selection is a “long‐listing” step that makes a first pass to decrease the list of potential features that might be used to separate the 2 cohorts. In this case, we used features associated with the deformation field to identify the morphological differences between a query dog and the reference dog. These deformation fields then can be grouped or clustered into 2 distinct subject types: a control group and a group with abnormal morphology. This was carried out using different features extracted from the images. The features used were the individual polar coordinates (ie, displacement and angle of displacement) of each of the MR image pixels that represent the deformation field, and the determinant of the Jacobian of the deformation field.^27^

To overcome any residual misalignments during the image acquisition process or associated with the image registration process, the deformation field was down sampled onto a coarser voxel grid before the feature extraction step. Because the degree of any potential misalignment was unknown, 3 different levels of downsampling were applied to the deformation field images: resampling at 3 × 3 pixels, 3.75 × 3.75 pixels and 5 × 5 pixels. This produced a smoothing effect on the deformation field, resulting in 3 downsampled data sets for CM‐P and 3 data sets for SM‐S. These were subsequently analyzed to understand the consistency of any features that might be found to separate the 2 types of subject (control and abnormal) morphology present.

### 2.4 Polar coordinates

The movement or displacement of a given pixel in the query image when registering to the reference image can be expressed in Cartesian (*x*,*y* coordinates) or polar coordinates. We chose to use the polar coordinate system, which represents the distortions or displacements of individual pixels as the magnitude of the displacement and the direction of displacement for each pixel, as opposed to its component deformation in the *x*‐ and *y*‐directions.^28^ This allows analysis of the magnitude or severity of deformations occurring within a specific area of the image, as well as the direction of these deformations, as separate entities.

### 2.5 Calculation of the Jacobian of deformations determinant

It was also desired to analyze the severity of the deformations in specific parts of the brain. In order to do so, the Jacobian of deformations was calculated. This technique is now commonly used in computational and imaging sciences. The Jacobian of deformations determinant is a measure of the expansion or shrinkage of local groups of voxels within an image region, wherein a matrix element value of 1 indicates no change for that voxel, a value <1 indicates shrinkage, and a value >1 indicates expansion.^27^

### 2.6 Data representation and noise reduction

Principal component analysis (PCA) was used to identify the most salient components in the data, facilitating the removal of less important components, such as noise or anomalies. Principal component analysis identifies combinations of feature components from the data that represent the major sources of variance and ranks these in order of saliency.^29^ Thus, the most salient principal components that describe 95% of the variance of the data were retained using PCA, whereas those from the remaining 5% were discarded as noise. These remaining components then were mapped back to the original image space.

### 2.7 Biomarker identification using machine learning

Initial identification of a broad set of potential candidate biomarkers was carried out using a machine learning technique known as feature selection. In the first experiment, we attempted to make an initial separation of dogs with CM‐P from those that do not have a history of behavior suggesting pain, wherein clinical examination by a veterinary neurologist did not find any evidence of spinal or head pain and where spinal MRI was reported as normal (CM‐N). In a second experiment, we attempted to distinguish between dogs that have clinically severe SM (SM‐S) and the controls (CM‐N).

### 2.8 Feature selection

The feature selection technique used in our study is known as sequential floating forward selection (SFFS)^30^, ^31^ and has been described previously.^32^ This process is applied to the deformation field, which is composed of a matrix that defines the distortion between the query image and the reference image. Each element of the matrix contains 2 values: the first is the magnitude of displacement between the corresponding pixels of the query image and reference image, and the second is the direction of this displacement. These 2 separate values are considered to represent separate features.

**Figure 3:**
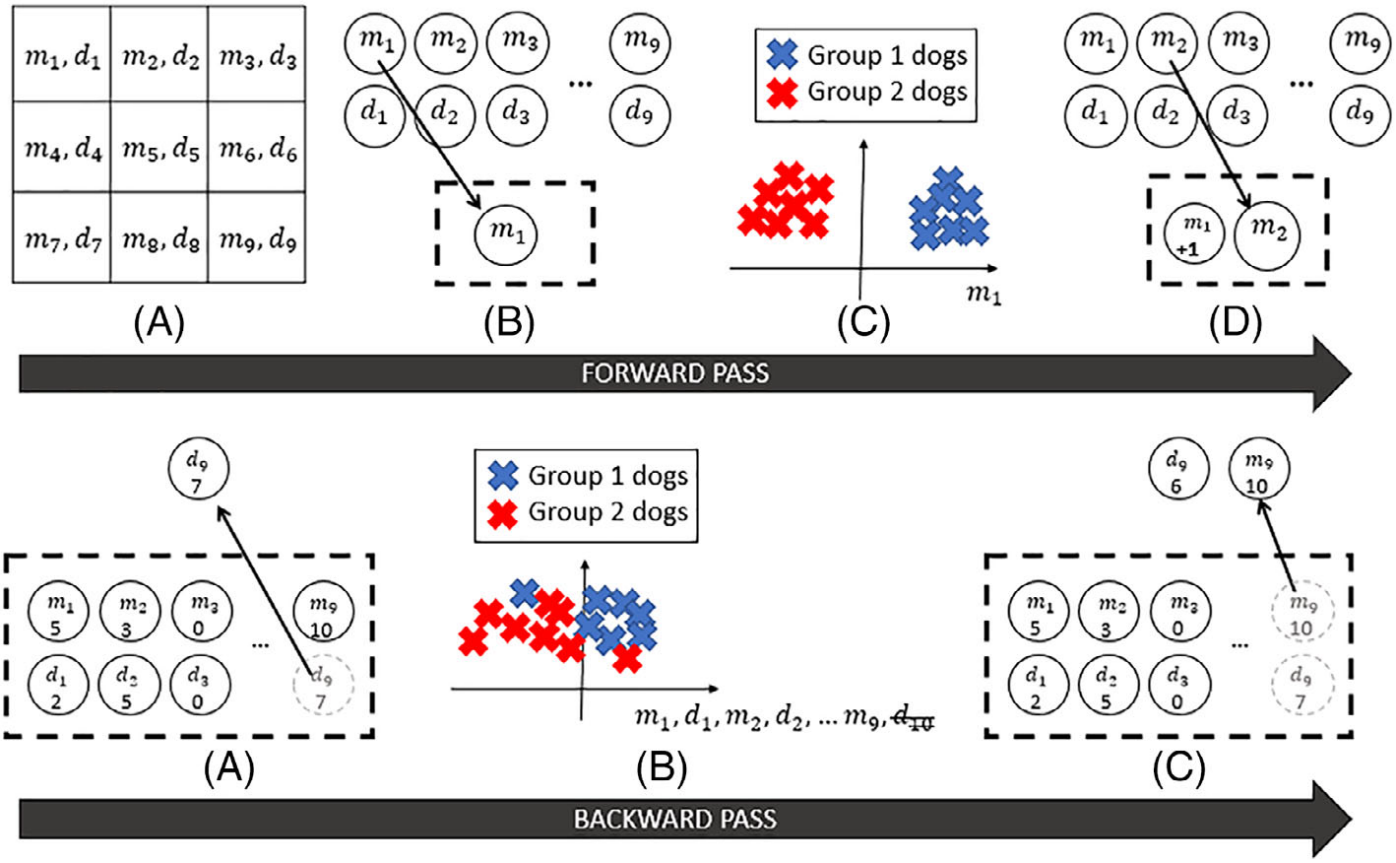
Schematic diagram of the sequential floating forward selection (SFFS) algorithm, which is split into 2 stages: the forward pass and the backward pass. In the forward pass: A, a simple 3 × 3 example of a deformation field which consists of pixel locations which each contain a displacement magnitude (mi) and a direction (di)—together these describe where a particular pixel is moved to during the image registration process that maps the query image onto the reference image. In the methodology presented here the direction and the magnitude are considered as separate features in the analysis; B, a single feature (either an “m” or a “d”) is randomly added to the feature subset or “longlist”; C, the feature subset is used to attempt a “first cut” separation of the dogs into 2 groups; D, the score(s) of this trial subset is increased if the grouping was successful (ie, produced better separation of the 2 groups), or decreased if unsuccessful. This forward pass is complete once all possible m's or d's have been added to the feature subset. Then, in the backward pass, A, the process is reversed wherein a single feature is randomly selected and removed from the subset; B, the feature subset is then used to separate dogs into 2 groups; C, the scores of feature removed from the subset is increased if the grouping was worse without it, or decreased if grouping was better without it, and a new feature is removed. The backward pass is complete when all the features are removed from the feature subset

Figure 3 shows a schematic of the SFFS algorithm, for which an example 3 × 3 sample of the deformation field is considered. The SFFS algorithm starts with an empty candidate feature subset (or list of features), and features are sequentially added to this subset. Initially, each feature has a score of 0. As each feature is added to the subset of features, the subset is tested to check whether this step was a useful step forward in classifying subjects into the 2 cohorts (controls, diseased). This is achieved using a simple classification method, known as k‐means clustering.^30^, ^31^, ^33^ If a newly added feature improves the k‐means clustering, then the individual feature's score is increased by 1. This initial process, known as forward pass, is repeated until all possible features have been tested for inclusion. In the second part of this algorithm, known as the backward pass, each feature is sequentially removed from the SFFS feature subset, and the dogs are reclassified into the 2 groups based on the features remaining in the feature subset. If the results improve, then this feature's score is decreased. On the other hand, if the classification results decrease, then this indicates that this feature was successful at distinguishing between the 2 groups and therefore its score is increased. The backward pass is performed to eliminate any redundant features from the optimal feature subset.

This process was repeated 250 times using different random orderings of features to account for any potential effects of ordering selection. Thus a set of candidate biomarkers was identified that contained the top 5 scoring features using SFFS.

### 2.9 Data classification

The final step in the methodology is classification, which attempts to optimally separate, or classify, the 2 groups of dogs (control, diseased). After the “long‐listing” step of feature selection to identify the most promising candidate features, we then used a state‐of‐the‐art classification algorithm to refine the way these features can be used to best separate the 2 cohorts of dogs. In this case, a kernelized support vector machine^34^ (SVM) was used as the classification method of choice because this approach has been shown to produce excellent results in cases of limited data size and where a complex or nonlinear decision may need to be made to separate 2 groups of subjects.^32^, ^34^

In order to assess the quality of the separation or classification method, ROC curves were calculated using the clinically defined labels (CM‐N vs CM‐P; CM‐N vs SM‐S) to estimate the true‐positive rate (TPR, also known as sensitivity) and false‐positive rate (FPR, also known as 1 − specificity) for the top 5 features (collectively).

In order to understand the potential variation of the neuromorphological patterns within the data, a process of cross‐validation was used. As part of this process, the data are split into 2 groups, a training set in which the SVM learns how best to separate control from SM/CM‐affected subjects, and a test set of previously unseen subjects from the original data set (same breed) that are used to test the performance of the classification process. In the first experiment (to analyze CM‐P separation), 5 randomly selected subjects from the entire data set of 26 cases were removed and considered as the test set. Thus, testing was undertaken on a set of images that the machine had not seen before. The SVM then was trained on the remaining 19 cases. The 5 test cases then were classified as part of the assessment. This process was repeated with >1000 combinations of training: test selections. The mean TPR and FPR were plotted for each, and the area under the curve (AUC) was computed. The variance also was recorded at each point to estimate the error. The same approach was taken for the second experiment (to analyze SM separation), with 5 subjects of the entire 18 cases excluded from the training process.

### 2.10 Biomarker mapping

The entire feature extraction and biomarker identification pipeline (Figure 1) was applied directly to the image pixels and to their respective deformation fields, each resampled at 3 different resampling rates. This resulted in 3 biomarker sets for the CM‐N vs CM‐P experiment, and 3 biomarker sets for the CM‐N vs SM‐S experiment. Each set of candidate biomarkers was mapped back to the reference image in order to visually demonstrate their location.

## 3 RESULTS

### 3.1 CM‐P analysis

**Figure 4:**
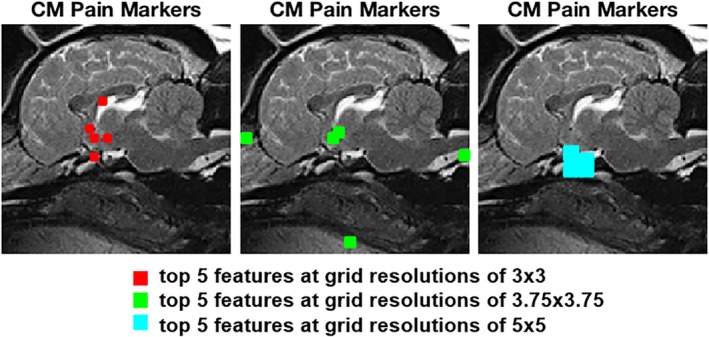
Image‐based biomarkers extracted from top 5 features at grid resolutions of 3 × 3 (red), 3.75 × 3.75 (green) and 5 × 5 (cyan) respectively for Chiari‐like malformation (CM) pain showing location on the reference control dog

The pipeline was first applied to an experimental data set consisting of 2 classes: a control group with CM (CM‐N) and a group diagnosed with CM‐P (CM‐P). The set of features that best separated morphological pathologies from CM‐N (as identified by the feature selection algorithm and validated by the SVM classifier) was mapped back onto the reference image identifying their anatomical locations. The 5 most relevant features identified by the SFFS algorithm, at 3 different resampling rates, are shown in Figure 4.

**Figure 5:**
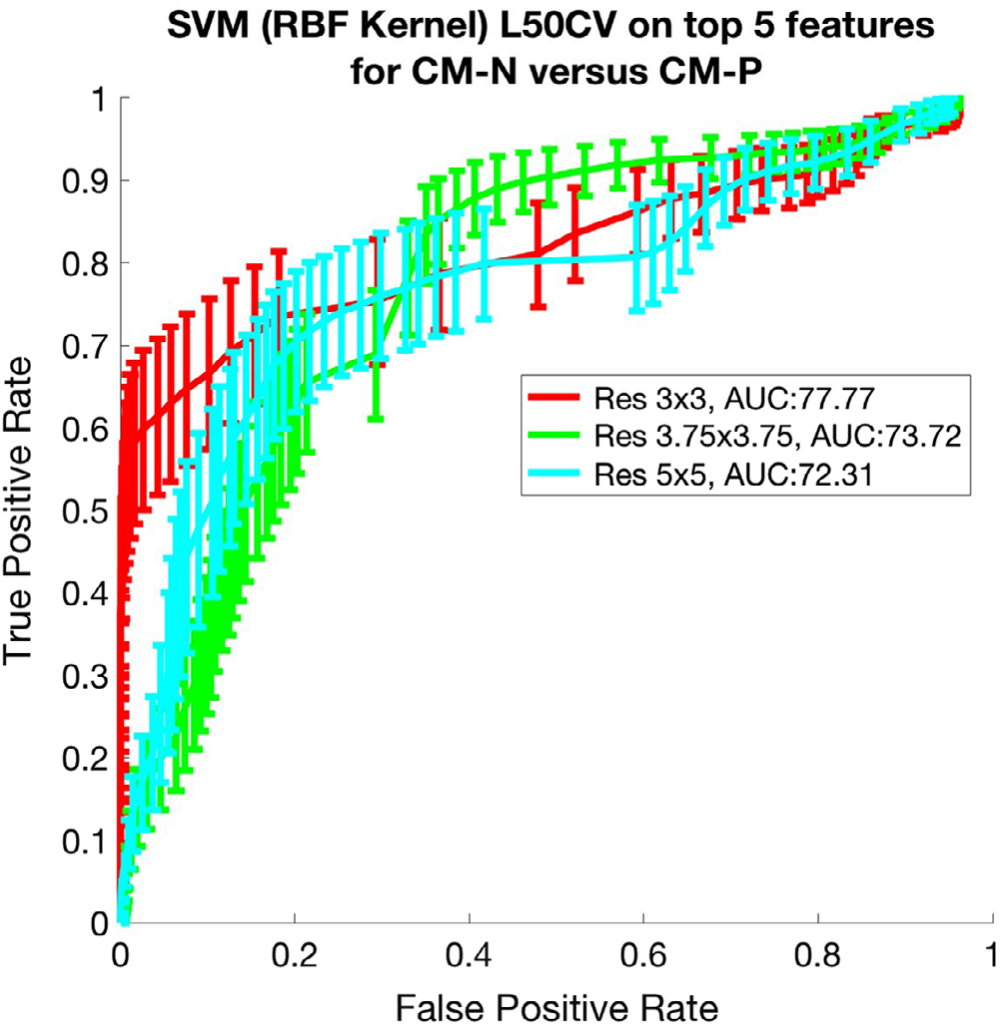
Graph showing receiver operating characteristic (ROC) Leave‐5‐out cross‐validation for the support vector machine (SVM) using the best 5 features selected using sequential floating forward selection (SFFS) to classify CM‐N vs CM‐P at different resampling rates. Area under the curve (AUC) metrics are shown in the legend

Figure 5 shows the corresponding ROC results for this experiment examining CM‐P separation, using the aforementioned “leave‐5‐out” cross‐validation approach. This approach refers to training the algorithm on all subjects within the data set except 5 subjects that are reserved for testing, and then changing the combinations of data within the training and testing data sets.

### 3.2 SM analysis

**Figure 6:**
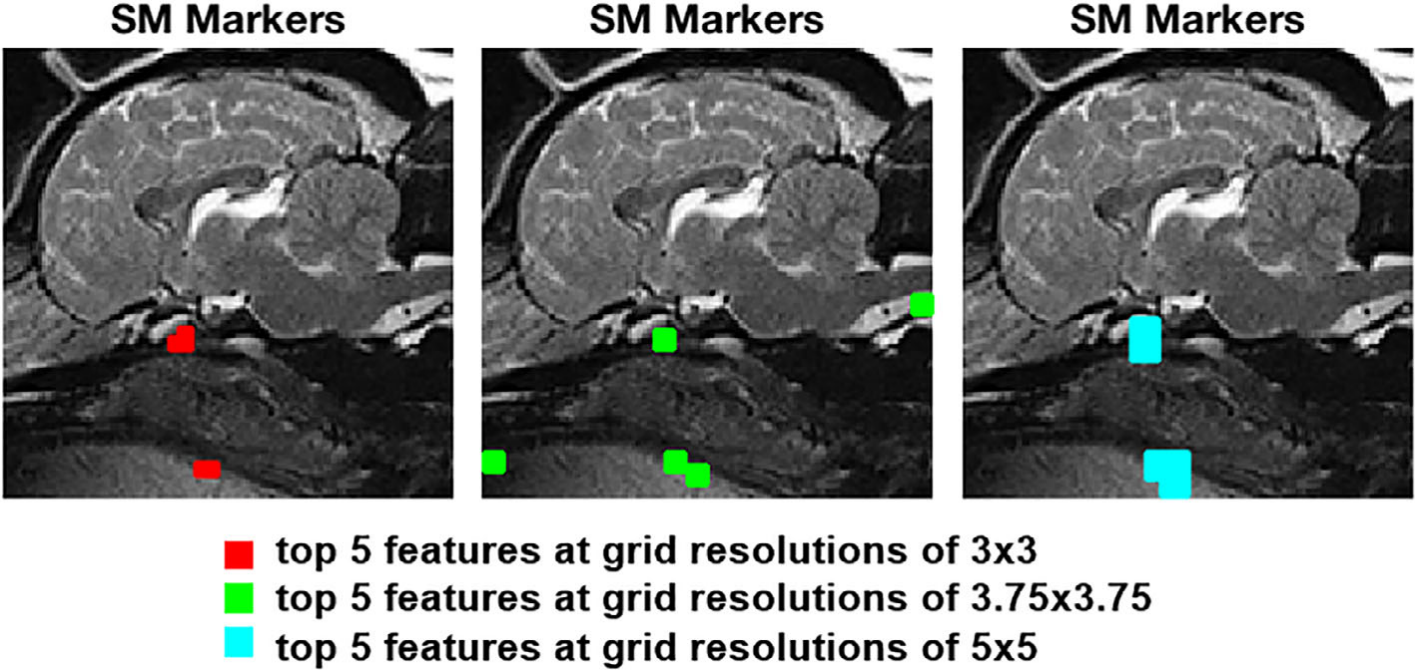
Biomarkers extracted from top 5 features at grid resolutions of 3 × 3 (red), 3.75 × 3.75 (green) and 5 × 5 (cyan) respectively for syringomyelia (SM)

**Figure 7:**
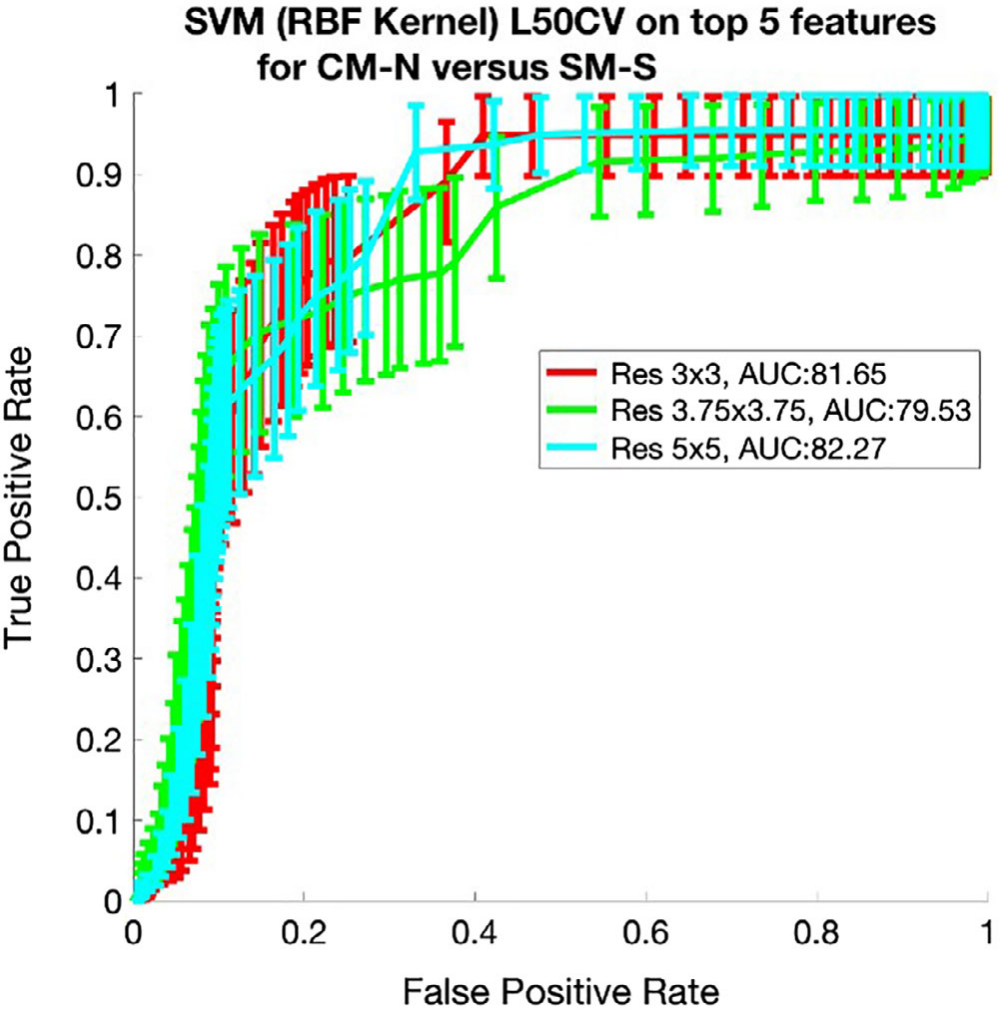
Graph showing ROC Leave‐5‐out cross‐validation for the SVM using the best 5 features selected using SFFS to classify CM‐N vs SM‐S at different resampling rates. AUC metrics are shown in the legend. AUC, area under the curve; ROC, receiver operating characteristic; SVM, support vector machine

Using the same approach as explained above, the following results were achieved for the data set consisting of 2 classes: CM‐N and SM‐S. Figure 6 shows the top 5 features identified by the SFFS algorithm for this data set, whereas Figure 7 shows the corresponding ROC curves exhibiting the separability between the 2 groups using these features.

## 4 DISCUSSION

We used a novel machine learning approach to produce a data‐driven analysis for identifying characteristic patterns associated with CM‐P and SM‐S in a midsagittal MRI slice. The advantage of this approach is that subtle characteristics in the image data can be identified that may not be immediately apparent on observation. The methodology developed here used a combination of image registration to identify neuromorphological differences associated with particular disease states, and machine learning to understand the location of the most important traits derived from the registration patterns, and identify when pathology was present or not. Although the method could be used in a variety of disorders, we have used CM‐P and SM‐S to illustrate the approach.

To allow for uncertainties and errors associated with variations in setup during MRI examination, as well as localized errors in the registration process, the associated deformation fields were downsampled to a coarser resolution, or grid, to smooth over minor inconsistencies. Thus, the analysis was undertaken over several levels of downsampling. Nonetheless, we demonstrated that the locations of many of the resulting key features (candidate biomarkers) were consistent, suggesting that the impact of downsampling was minimal. The discriminatory performance of these candidate biomarkers was assessed by ROC analysis. Anatomical features of interest can be observed in Figure 5 (CM‐N; CM‐P) and Figure 7 (CM‐N; SM‐S).

### 4.1 CM‐P analysis and clinical relevance

The AUC for the ROC curves that were plotted to validate CM‐P features is shown in Figure 6, with AUC values up to 0.78 and a mean value of 0.74. These ROCs suggest an attainable operating point with a sensitivity of 82% and a specificity of 69%.

In Figure 4, several clusters of features have been identified: the markers plotted in red are located in the area of the rostral wall (lamina terminalis) and floor of the third ventricle, optic chiasm and corpus callosum; the markers plotted in green are located within the caudal nasal cavity close to the dorsal cribriform plate, between the soft palate and the tongue, on the tip of the odontoid process and in the floor of the third ventricle; the markers plotted in cyan are located in the sella turcica and the sphenoid bone complex (presphenoid and basisphenoid).

A cluster in the region of presphenoid bone and the optic chiasm immediately rostral to the sella turcica is clinically consistent. Early closure of the basispheno‐presphenoid synchondrosis and therefore a shortened presphenoid bone has been associated with brachycephaly and airorhynchy. Early fusion of the basisphenoid presphenoid synchondrosis is a feature in mouse models of Crouzon syndrome, which is a complex craniosynostosis syndrome that can be associated with Chiari type I malformation and which has some similarities to CM in dogs.^35^

Features of the corpus callosum and third ventricle may be related to the ventriculomegaly that often is associated with CM.^23^ As the lateral and third ventricles become dilated, the corpus callosum becomes thinner and more dorsally elevated. Another group of features was identified between the soft palate and the tongue. There are 2 possible explanations for a link between CM‐P and a soft tissue structure that is outside the nervous system. The first is brachycephaly or airorhynchy as the shortening of the bones is not mirrored by a reduction in the cranial soft tissue structures. Thickening of the rostral and lengthening of the caudal soft palate are typical anatomical abnormalities associated with this condition especially in the CKCS.^36^ It has been shown previously that CM‐P is associated with brachycephaly and soft palate changes may have a coincidental association. The second explanation is that brachycephalic obstructive airway syndrome (BOAS) secondary to conformational changes in the soft palate may predispose CM‐P. For example, increased expiratory effort because of increased airway resistance (Valsalva) may alter CSF dynamics, venous pressure or both. Whether CKCS with CM‐P are more likely to suffer BOAS or vice versa has not been established and represents a new area of enquiry. There was also a single marker in the caudal nasal cavity close to the cribriform plate. Finally, there was also a single biomarker at the tip of the odontoid process. It has been recognized previously that dogs with CM and SM may have craniocervical junction abnormalities including rostral displacement of the axis and atlas with increased odontoid angulation causing craniospinal junction deformation and medulla oblongata elevation.^4^, ^8^

### 4.2 SM analysis and clinical relevance

The features related to SM were consistent over the 3 different resampling rates. Figure 6 shows that the main features related to SM were found to be located in the same areas for all 3 different downsampled images: the markers plotted in red are located in in the presphenoid bone in the optic canal region just cranial to the sella turcica together with a single marker between the soft palate and tongue; the markers plotted in green are located in the optic canal region just cranial to the sella turcica in the presphenoid bone, on the hard palate and between the soft palate and the tongue and at the tip of the odontoid process; and the markers plotted in cyan are located just cranial to the sella turcica in the presphenoid bone together with a single marker between the soft palate and tongue.

From the comparison of the locations of the top scoring features related to CM‐P with those related to SM, it is evident that both conditions are associated with a common feature of the presphenoid bone. Other CM markers in the brain are dorsal to this region, and there is also a marker common to CM and SM in the soft palate ventral to this bone.

In the case of SM classification, for the ROCs produced by combining the results of the top 5 features into the classifier, shown in Figure 7, AUC values up to 0.82 with a mean value of 0.81 were obtained. This suggests an operating point with a sensitivity of 93% and a specificity of 67% is attainable.

### 4.3 Limitations of study

The image registration process may be prone to errors because the mid‐sagittal slices of each MRI scan were not aligned because of differences in orientation of the dog within the MRI scanner. Furthermore, the machine learning techniques used in our study were chosen to suit a smaller data set, but a larger data set of dogs may allow the use of more sophisticated techniques and provide further insight into CM‐P and SM. Another limitation was the small number of normal dogs and the fact that the reference average subject was used as normal for all comparisons. It is not known whether this subject would reflect the average for the entire population.

### 4.4 Future study and software availability

Future directions for research should aim at improving the registration process in order to address the difference in the dog's orientation during image acquisition so as to produce a more robust pipeline. The clinical implications of our findings should be explored further to try to understand the underlying mechanisms of CM associated pain and SM. As part of a future study, it would be interesting to compare the discriminability of the identified markers to that of traditional markers of CM and SM, as well as to test the discriminability of these markers on dogs from different pedigrees and origin.

The methodology described here was developed using in‐house software based on MATLAB—a specialist software development environment for scientific research purposes. Because ours was a proof‐of‐concept study, the associated software is not currently suitable for routine use, but the techniques described here potentially could form part of a commercial product after further development.

## 5 CONCLUSION

We evaluated a fully automated pipeline for the identification of potential biomarkers in CKCS dogs with SM and CM associated pain, with the aim of expanding the knowledge base of these disorders. From the comparison of dogs with CM‐P with the control group, the main regions identified as diagnostically relevant candidate biomarkers for this condition are the floor of the third ventricle and closely associated neural tissue, and the region in sphenoid bone (presphenoid and basisphenoid) around the sella turcica. There are also lesser areas of interest in caudal nasal cavity close to the dorsal cribriform plate, between the soft palate and the tongue, on the tip of the odontoid process.

The main regions identified as diagnostically relevant biomarkers when comparing dogs with a specific diagnosis of SM to a control group were the presphenoid bone and the region between the soft palate and the tongue. Both experiments have yielded biomarkers in the presphenoid bone and the area between the soft palate and the tongue, which indicates both conditions being strongly related to changes within this area. Further work is needed to explore the further development of the image registration process, which in turn is expected to improve the AUC values.

We were able to successfully discriminate between CM‐P subjects and those without, and for those with SM‐S, and those without, using a set of biomarkers discovered using machine learning. This work can be used as a basis to build a clinical diagnostic test based on registering these key locations in a query subject back to the equivalent landmarks on a reference subject free of such pathology. Further analysis could be done to investigate whether a link exists between disease severity and the magnitude of the neuromorphological distortion indicated by these biomarkers.

## CONFLICT OF INTEREST DECLARATION

Authors declare no conflict of interest.

## OFF‐LABEL ANTIMICROBIAL DECLARATION

Authors declare no off‐label use of antimicrobials.

## INSTITUTIONAL ANIMAL CARE AND USE COMMITTEE (IACUC) OR OTHER APPROVAL DECLARATION

This retrospective study analyzed Digital Imaging and Communications in Medicine (DICOM) data obtained from dogs that underwent MRI either for diagnostic purposes for assessment of CM/SM status before breeding or for diagnostic investigation of neurological signs, pain, or both.

## HUMAN ETHICS APPROVAL DECLARATION

Authors declare human ethics approval was not needed for this study.
